# Outpatient opioid prescribing by Alzheimer’s diagnosis among older adults with pain in United States

**DOI:** 10.1186/s12877-023-04115-6

**Published:** 2023-08-01

**Authors:** Yinan Huang, Rajender R Aparasu, Tyler J Varisco

**Affiliations:** 1 Department of Pharmacy Administration, University of Mississippi College of Pharmacy, 235 Faser Hall, Oxford, Mississippi, 38677 USA; 2 Department of Pharmaceutical Health Outcome and Policy, University of Houston College of Pharmacy, Houston, USA; 3 Prescription Drug Misuse Education and Research Center, University of Houston College of Pharmacy, Houston, USA

**Keywords:** Opioid prescribing, Older adults, Pain management, Prescribing pattern, Alzheimer’s diseases

## Abstract

**Objective:**

To examine opioid prescribing practices for pain in older adults with and without Alzheimer’s Disease and Related Dementias (ADRD).

**Methods:**

This cross-sectional study used National Ambulatory Medical Care Survey data (2014–2016, and 2018). Adults aged ≥ 50 years with pain were analyzed. Prescribing of opioid and concomitant sedative prescriptions (including benzodiazepines, Z-drugs, and barbiturates) were identified by the Multum lexicon code. Multivariable logistic regression evaluated the risk of opioid prescribing or co-prescribing of opioid and sedative associated with ADRD in older adults with pain.

**Results:**

There were 13,299 office visits in older adults with pain, representing 451.75 million visits. Opioid prescribing occurred in 27.19%; 30% involved co-prescribing of opioids and sedatives. ADRD was not associated with opioid prescribing or co-prescribing of opioid and sedative therapy.

**Conclusions:**

Opioid and sedatives are commonly prescribed in older adults with pain. Longitudinal studies need to understand the etiology and chronicity of opioid use in older patients, specifically with ADRD.

## Highlights

This national study examined the opioid prescribing practices for pain in older adults with and without Alzheimer’s Disease and Related Dementias (ADRD).

The study found about 30% of visits for older adults with pain resulted in opioid prescriptions, and the co-prescribing of sedatives were noted in 30% of these visits made by older adults with pain where an opioid were prescribed.

No significant difference exists between ADRD vs. non-ADRD groups in receiving opioid prescriptions or co-prescription of opioids and sedatives among the visits with pain.

Future research should aim to understand the health outcomes associated with opioid prescribing and concomitant receipt of sedatives in ADRD.

## Introduction

Opioids are commonly, although somewhat inappropriately, used to manage pain in older adults. Approximately 20% of US population filled at least one opioid prescription in 2018, with the highest opioid prescribing rate in older adults [1]. Opioid use in older adults may increase risk of delirium, falls, and fracture. The American Geriatrics Society (AGS) Beers Criteria strongly recommends against the use of opioids in older adults. Moreover, benzodiazepines and related hypnotics (Z drugs), often referred to as BZDRs, should also be used cautiously in older people, especially those with underlying Alzheimer’s Disease and Related Dementias (ADRD) as they may exacerbate cognitive impairment and increase risk of serious adverse events, including risk of fractures/falls, and hospitalizations [2].

The AGS Beers Criteria suggests to avoid the use of BZDRs in older patients and strongly recommends against the concomitant use of Benzodiazepines and opioids [3]. Despite this consensus recommendation and the risk, opioids and BZDRs are prescribed concomitantly in as many as nearly 1 in 3 older people with ADRD [4]. The objective of this study, was therefore, to examine patterns of opioid prescribing among older adults with pain and to compare the patterns of opioid prescribing among those with ADRD vs. those without. The study also evaluated the concomitant use of sedative prescriptions and opioids among those older adults with and without ADRD.

## Methods

### Data source and study design

The retrospective cross-sectional study was conducted using National Ambulatory Medical Care Survey (NAMCS) data from 2014-2016, and 2018, a publicly available dataset containing records of visits to outpatient facilities in the United States. NAMCS is a national, annual survey, administered by the CDC, that samples outpatient visits to non-federal, office-based providers at community health centers across the US [5]. NAMCS employs a multi-stage probability sampling design to identify a sample of providers capable of representing all providers in the US. At each sampled visit of healthcare professionals, standardized survey collected data about the office visits related to patient care, such as the reason for encounter visits, medical diagnoses coded in ICD-9 or ICD-10 CM codes, medications ordered, patients’ demographics, and providers’ characteristics and practice. Each study record captures up to thirty medications prescribed or used at baseline at each visit. These are classified using the Multum Lexicon system to enable researchers to characterize medication use. After data is collected, complex sampling weights based on patterns of healthcare utilization in the US are used to upsample visits with characteristics that are under-represented and to downsample those with characteristics that are over-represented in the unadjusted dataset. The study was approved under the exempt category by the University of Houston Institutional Review Board.

### Study sample and outcome measures

This study identified all office visits involving adults at least 50 years of age with a condition likely to cause pain. These were defined using a list of ICD-9 and ICD-10 codes for painful conditions including (a) Abdominal pain, (b) Back, head & neck pain, (c) Fibromyalgia, neuropathy & systemic disorders related pain, (d) Fractures, contusions, sprains and strains, and (e) Limb/extremity pain, joint pain and non-systemic, non-inflammatory arthritic disorders. Visits made by patients with these conditions were identified based on a series of ICD-9-CM and ICD-10-CM codes. The primary measure of exposure was ADRD status. ADRD diagnosis was operationally defined using a binary indicator variable for clinicians who reported Alzheimer’s disease from the visit questionnaire. Additional ADRD visits were captured through ICD-9-CM code (290, 291.2, 294, 331, 797) and ICD-10-CM codes (G30.0, G30.1, G30.8, G309, F01.50, F01.51, F02.80, F02.81, F03.90, F03.91, F10.27, F10.97, F13.97, F18.27, F18.97, F19.27, F19.97) [6, 7].

The outcome of interest was receipt of an opioid prescription. All opioid prescriptions were operationally classified as narcotic analgesics and narcotic analgesic combinations, and identified using Multum database Lexicon Plus in terms of therapeutic classes drug category codes, in which the narcotic analgesics and narcotic analgesic combinations coded as ‘060’ and ‘191’, respectively [8, 9]. Use of benzodiazepines, Z-drugs and barbiturates, jointly classified as use of sedative medications, was also identified for each visit using the Multum therapeutic classification system. Visits were classified as opioid alone or concomitant opioid and BZDR visits.

### Statistical analysis

Descriptive analyses, adjusted for the complex survey design, were conducted to characterize outpatient visits by older people with pain. The weighted analyses were also performed to estimate the overall opioid use as well as concomitant use of sedative prescriptions (benzodiazepines, Z-drugs and barbiturates) and opioids, considering the complex survey design and adjusting for the variables of the cluster, strata and weight. Two weighted, multivariable logistic regression models, adjusted for pain diagnosis, comorbidities, and patient demographics were used to measure the association between ADRD diagnosis and receipt of opioid monotherapy and opioid and BZDR combination therapy, respectively. All analyses were conducted in SAS 9.4 (SAS Institute, Cary, North Carolina).

The Andersen Behavioral Model, a commonly used conceptual framework in health services research, was employed to guide the selection of covariates and to conceptually classify them as predisposing, enabling, and need characteristics that are known to contributed to health services utilization. [10] Variables categorized into these domains were selected based on the published literature and available data in the NAMCS.[11–13] Predisposing characteristics included age, sex, race, ethnicity, metropolitan statistical areas. Enabling characteristics included US Census region of residence, payer type, physician specialty and insurance typ. Need characteristics included year of visit, Elixhauser comorbidity score, and substance use disorders &psychiatric conditions, reasons for visit [14].

## Results

There were 13,299 unweighted visits for older adults with a painful condition, nationally representing a total number of 451.75 million (SE: 23.31 million) outpatient visits in the US during the study period for an annual mean (SD) of 112.94 (5.83) million visits. Of the national sample of older adults with pain, 1.42% of visits were also related to ADRD, nationally representing 1.60 million (SE: 0.43 million) visits each year.

Table 1 shows the characteristics of outpatient visits among these older adults with pain, by AD status. Some baseline characteristics were comparable between ADRD and non-ADRD groups, including demographics of sex, race, ethnicity, region, and clinical characteristics of reasons for visits and certain type of painful conditions (such as limb/extremity pain, back pain, fibromyalgia). However, significant differences were noted between those with ADRD versus those without ADRD in age group, metropolitan location, payment source, provider specialty, and survey year. Also, compared to non-ADRD visits, those with ADRD were more likely to have fractures and less likely to have abdominal pain. In addition, those with ADRD were more likely to have substance use disorders than their non-ADRD counterparts.

**Table 1:** Study Characteristics. Outpatient Visits Among older adults with pain, by ADRD status From the US NAMCS

Characteristics	ADRD			Non-ADRD			P-value
	Visits (N = 150)	Weighted Visits (N = 6,394,296)	Percent (%)	Visits (N = 13,149)	Weighted Visits (N = 44,535,9925)	Percent (%)	
***Predisposing Characteristics***							
**Age**							
50–64	13	512,215	8.01	6483	218,598,665	48.39	**< 0.0001**
65–74	37	1,351,901	21.14	3853	126,850,292	28.08	
75–84	50	1,546,422	24.18	2101	71,575,917	15.84	
85+	50	2,983,758	46.66	712	28,335,052	6.27	
**Sex**							
Female	93	3,946,849	61.72	7618	267,139,830	59.98	0.7973
Male	57	2,447,447	38.28	5531	178,220,095	40.02	
**Race**							
White	130	5,293,107	82.78	11,448	363,152,360	80.39	0.773
Black	16	796,173	12.45	1220	45,947,754	10.17	
Other [1]	4	305,016	4.77	481	36,259,812	8.03	
**Ethnicity**							
Hispanic/Latino	13	1,193,849	18.67	971	47,895,758	10.6	0.2594
Non-Hispanic/Latino	137	5,200,447	81.33	12,178	397,464,167	87.98	
**Region**							
Northeast	13	832,897	13.03	2049	75,302,779	16.91	0.8936
Midwest	52	726,753	11.37	3477	67,056,373	15.06	
South	45	2,265,874	35.44	4115	142,062,444	31.9	
West	40	2,568,771	40.17	3508	160,938,329	36.14	
**Metropolitan Statistical Area**							
Yes	136	6,217,156	97.23	11,742	406,883,671	90.07	**0.0036**
No	14	177,140	2.77	1407	38,476,255	8.52	
***Enabling Characteristics***							
**Payment Source**							
Medicare & Medicaid	110	5,253,094	82.15	6625	229,834,200	51.61	**0.0005**
Other [2]	20	452,194	7.07	1412	44,478,131	9.99	
Private insurance	20	689,008	10.78	5112	171,047,595	38.41	
**Provider specialty**							
Primary care specialty	95	5,195,066	81.25	4510	192,704,326	43.27	**< 0.0001**
Surgical care specialty	22	478,508	7.48	5266	108,539,604	24.37	
Medical care specialty	33	720,722	11.27	3373	144,115,995	32.36	
**Survey year**							
**2014**	83	1,282,544	20.06	7589	151,179,224	33.46	**0.005**
2015	52	4,210,304	65.84	4205	172,603,030	38.21	
2016	7	505,406	7.9	791	55,553,723	12.3	
2018	8	396,042	6.19	564	66,023,948	14.62	
***Need characteristics***							
**Reason for visits**							
Established case	134	5,312,074	83.08	10,632	380,115,732	85.35	0.7676
New case	16	1,082,222	16.92	2517	65,244,193	14.65	
**Type of Painful conditions**							
**Limb/extremity pain, joint pain and non-systemic, non-inflammatory arthritic disorders**							
No	85	3,804,139	59.49	8062	275,658,445	61.9	0.84
Yes	65	2,590,158	40.51	5087	169,701,480	38.1	
**Back, head & neck pain**							
No	105	4,122,674	64.47	9206	305,806,637	68.67	0.5315
Yes	45	2,271,622	35.53	3943	139,553,288	31.33	
**Abdominal pain & other painful conditions**							
No	120	5,691,150	89	9100	320,426,423	71.95	**0.0149**
Yes	30	703,146	11	4049	124,933,502	28.05	
**Fibromyalgia, Neuropathy & Systemic disorders, or diseases causing pain**							
No	124	5,585,906	87.36	11,168	359,609,479	80.75	0.2489
Yes	26	808,390	12.64	1981	85,750,446	19.25	
**Fractures, contusions, sprains, and strains**							
No	126	4,421,547	69.15	10,623	372,130,380	83.56	**0.0471**
Yes	24	1,972,749	30.85	2526	73,229,546	16.44	
**Substance use, anxiety, & psychiatric disorder**							
No	145	5,495,745	85.95	12,908	426,487,977	95.76	**0.0387**
Yes	5	898,551	14.05	241	18,871,948	4.24	
	Mean [SD]				Mean [SD]		
**Elixhauser Index**	1.64 [0.24]				0.73 [1.57]		

### Patterns of opioid prescribing and co-prescribing of opioids and sedatives

Table 2 provides prescribing practice related to opioid prescription or co-prescribing of opioids and sedatives during office visits by older adults with pain. Overall, of all these sampled outpatient visits, 27.19% resulted in an opioid prescription, with an estimated 30.70 million (SE: 2.36 million) opioid prescription annually. Sedative prescriptions were co-prescribed in almost 30% of these visits made by older adults with pain receiving an opioid prescription.

**Table 2:** Multivariable logistic regression of adjusted association results. Opioid Prescribing & Concomitant Opioid and Sedatives Use, by AD Status Among Elderly Patients Involving a Painful Condition in NAMCS, 2014–2016, 2018

Model 1: Outcome = Prescribed Opioid Prescription		
	Adjusted OR ¥	95% CI
**ADRD Status**		
**Yes**	1.36	0.80–2.30
**No**	1 [Reference]	
**Model 2: Outcome = Prescribed Opioid Prescription in combinations with BZDRs**		
	**Adjusted OR $**	**95% CI**
**ADRD Status**		
**Yes**	1.84	0.83–4.06
**No**	1 [Reference]	

Abbreviations: ADRD, Alzheimer’s disease; OR, odds ratio; CI, confidence interval; BZDR, benzodiazepines and benzodiazepine-related drugs.

¥ Adjusted for demographics and clinical characteristics listed in Table 1

$ Adjusted for demographics and clinical characteristics listed in Table 1

### Association of ADRD status with opioid prescription

Table 2 shows the effect of interest from two, adjusted multiple logistic models evaluating the f association between ADRD status and receipt of opioids and concomitant opioid and BZRD medications, respectively. The first model examining the association between ADRD status and receipt of opioid monotherapy found no association between ADRD status and medication receipt. (aOR: 1.356 (0.798–2.302)) (Table 2). Similarly, ADRD status was not associated with receipt of combination opioid and BZRD treatment(aOR: 1.836 (0.831–4.056)).

## Discussion

We found that adults with ADRD are neither more nor less likely than those without ADRD to be prescribed opioids or opioid and sedative combination therapy at US outpatient visits. In this multi-year, nationally representative sample, more than one-quarter of all outpatient office visits for older adults resulted in an opioid prescription and over 30% of these visits ended in sedative co-prescription. The fact that opioid prescribing was not clearly associated with ADRD status suggests that ADRD is not an independent predictor of opioid prescribing. These findings contrast with an earlier study in Medicare data saying that patients with ADRD were less likely than those without to receive an opioid prescription [11]. Yet another US study using Medicare claims and another European study, however, found that ADRD patients were more likely to receive opioid prescriptions [12, 13]. The lack of consensus in this area suggests that more, rigorous longitudinal work is still needed to understand predictors and outcomes of the common use of this potentially dangerous combination of medication in older adults with ADRD. We limited our analysis to those with a comorbid painful condition-those with an increased baseline likelihood of receiving an opioid prescription. Our results demonstrate that providers often look past potential cognitive decline and increased fall risk and still freely prescribe opioids to individuals with ADRD.

The lack of association between ADRD status and opioid prescribing is highly concerning and raises questions about the quality of pain management in individuals with ADRD. In a recent matched cohort study, Taipale and colleagues found that incident opioid use doubles the risk (aHR: 1.96) of fall and fracture in older adults with Alzheimer’s disease [15]. Risk of fall was highest within the first two-months of opioid use (aHR: 2.27) and varied with opioid potency [15]. Therefore, more work is needed to identify differences in trajectory of opioid pharmacotherapy and the incidence of adverse, clinically relevant outcomes in older adults with and without ADRD. There is also a need for continuous evaluation of the extent of co-prescribing of these high-risk medications in the vulnerable ADRD patient group. Future research needs to focus on understanding the sequelae of opioid and sedative combination use in older adults with ADRD. Overall, there is a strong need for concerted efforts to optimize opioid prescribing in ADRD group.

Overall, this study found no variation in the use of opioid medications between the group with or without ADRD. Tailored, pain management guidelines for patients with ADRD are lacking and our findings suggest that providers manage pain in patients with memory loss not differently in prescribing opioids than they do for other older adults. The lack of differences in opioid prescribing speaks fundamentally to the well document challenges providers face in assessing pain among older adults with ADRD [16]. Although many patient reported pain measurement tools exist, these are poorly validated in older adults with ADRD [17]. In addition, there are organizational shortcomings in the care setting often hamper the quality of care, including pain management in the ADRD group [16]. For example, in a survey of nurses and other health workers in European long-term care settings, a lack of education in pain management among the nurses and healthcare workers was reported [18]. In a recent semi-structured interview of nurses in 12 nursing homes in Sweden, nurses described the communicative and organizational challenges when they provided care for the advanced dementia persons with pain, highlighting a demanding need for training and educating skilled nursing facilities to improve the quality of care among people with dementia and pain [19]. Therefore, to provide effective care in pain management in the ADRD population, concerted efforts and practical insights from a multidisciplinary team are needed. Preventing adverse sequelae of opioid pharmacotherapy in older adults with ADRD requires that caregivers at every point in the process of care, including family caregivers, must be trained to identify and appropriately manage pain alongside other conditions, especially in the setting of BZRD pharmacotherapy.

### Strengths and limitations

The use of a recent, representative, national database allowed us to characterize current use of opioid and BZRD agents in office based visits for older adults with and without ADRD. In spite of these strengths, this current analyses had several limitations. As NAMCS surveys involve physician practice in nonfederal office-based patient care, current findings are not generalizable to institutionalized patients. Furthermore, visits to hospital-based clinics may be under-represented in NAMCS.

In addition, the NAMCS is a series of cross-sectional surveys which is representative of visit-level data, not patients. This limits the ability to make population based inferences and makes it impossible to establish causality. Furthermore, NAMCS does not collect detailed information on pain severity making it impossible to determine if opioid use was associated with more severe pain. Likewise, there was lack of information of the duration of utilization of opioid pain medications. NAMCS does not contain measures of socioeconomic status, prescriber preference, or prescriber training outside of specialty certification meaning that it is difficult to fully operationalization all constructs of the Andersen Behavioral Model. Lastly, residual confounding may have resulted in statistical imbalance between ADRD vs. non-ADRD group. The cross-sectional nature of the data source makes it difficult to examine this through mediation and moderation modeling. This may have increased the risk of type 1 error, leading us to conclude that there was no association between ADRD status and opioid prescribing. Simply put, the unbalance distribution between the ADRD vs. non-ADRD group may explain the insignificance observed in the results: since there are not many people in the ADRD group, the number of patients with the opioid outcome is also less in the ADRD group, causing a large standard deviation and hence leading to a wide confidence interval of the adjusted odds of opioids associated with ADRD group. Future longitudinal studies using a large database are needed to validate the results of this analysis. In light of these limitations, these results still contribute to our collective understanding of the types of healthcare visits that lead to opioid prescribing in ADRD and non-ADRD patients.

## Data Availability

The datasets generated and/or analysed during the current study are available in the [Centers for Disease Control and Prevention], [National Center for Health Statistics]. For more information, check: https://www.cdc.gov/nchs/ahcd/datasets_documentation_related.htm.
